# Controlled encapsulation and droplet size prediction in two-step microfluidic double emulsions

**DOI:** 10.1039/d6lc00406g

**Published:** 2026-06-24

**Authors:** Chen Tang, Loïc Chagot, Panagiota Angeli

**Affiliations:** a ThAMeS Multiphase, Department of Chemical Engineering, University College London UK p.angeli@ucl.ac.uk; b TBI, Université de Toulouse, CNRS, INRAE, INSA Toulouse France

## Abstract

Double emulsions of water-in-oil-in-water (w_1_/o/w_2_) are critical for encapsulation and controlled release in microfluidic applications. In this work, double emulsion droplets were produced *via* a two-step flow-focusing process in a microchannel. A low-viscosity silicone oil (4.6 mPa s) was used as the oil phase, while the aqueous phase consisted of 48 wt% water and 52 wt% glycerol. Sodium dodecyl sulfate (SDS) was added at concentrations 0.2, 0.5, 1, and 2 times its critical micelle concentration (CMC = 11 mM) to systematically vary the interfacial tension. Three distinct formation regimes, namely drop-in-drop, drop-in-plug, and drop-in-thread, were identified under varying flow rate ratios and interfacial tensions. A semi-empirical model was developed to predict double droplet size in the drop-in-drop regime, which has significant practical relevance. For conditions with *C*/CMC ≥ 1, where interfacial tension is assumed to have reached equilibrium, good agreement with the experimental data was obtained, with mean absolute percentage errors (MAPE) of 8.16% for the core droplets and 9.54% for the double droplets. For lower surfactant concentrations, incorporating dynamic interfacial tension gave a MAPE of 8.17% for double droplet size prediction. These results provide a quantitative framework for droplet size prediction and offer operating guidance for the controlled generation of encapsulated droplets in microfluidic systems.

## Abstract 2

Water-in-oil-in-water double emulsions were generated using a two-step microfluidic flow-focusing device. The proposed semi-empirical model predicts droplet sizes, providing practical guidance for controlled encapsulation.

## Graphical abstract

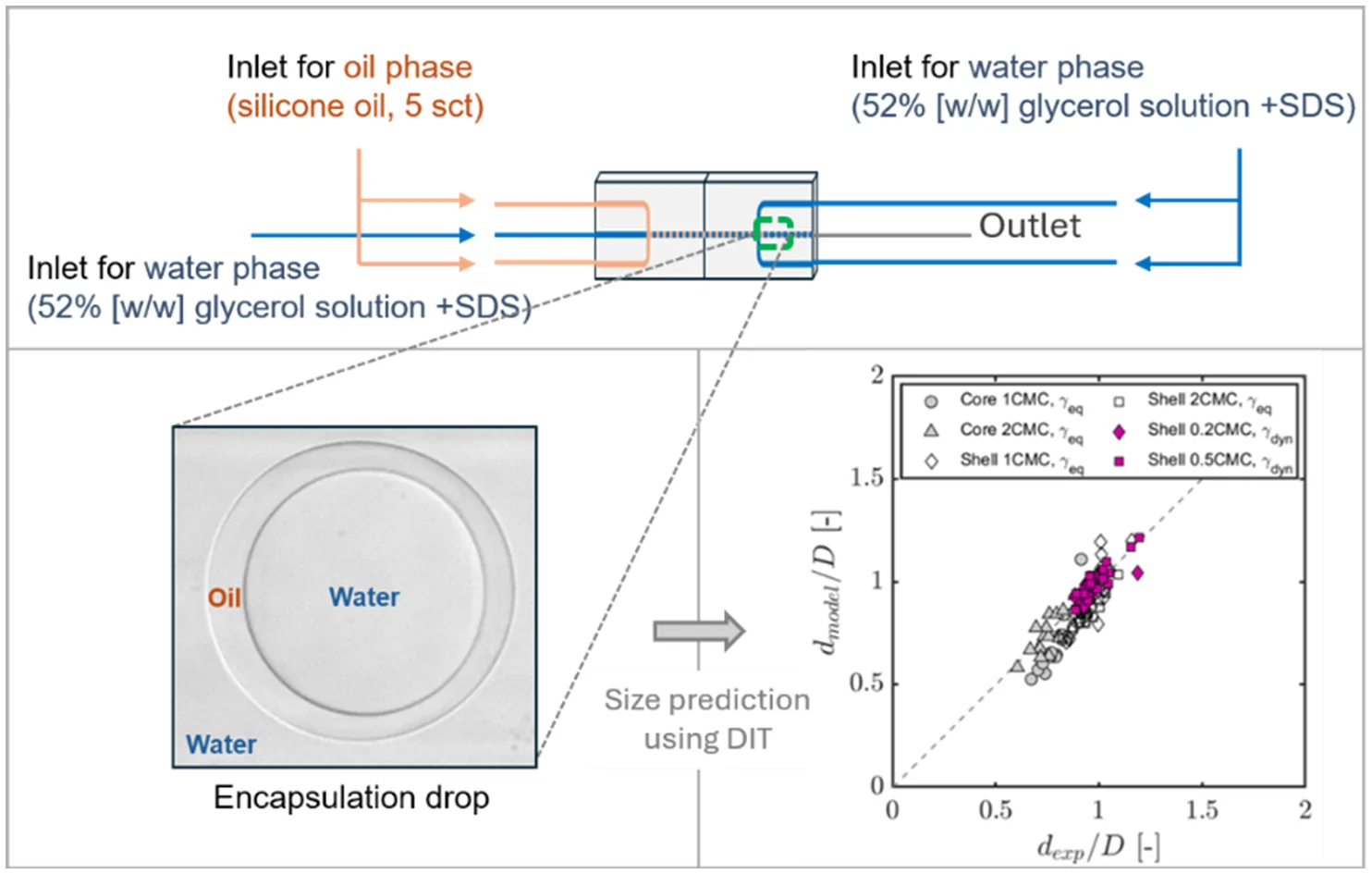

## 1 Introduction

Double emulsion droplets, such as water-in-oil-in-water (w_1_/o/w_2_) and oil-in-water-in-oil (o_1_/w/o_2_) are important core–shell systems for encapsulation, compartmentalization, and controlled release of materials, with broad relevance in drug delivery,^1,2^ biotechnology,^3,4^ particle fabrication^5–7^ and microreaction technology.^8^ Their hierarchical structure allows functional ingredients to be incorporated into distinct phases and enables control over interfacial properties.^9,10^ As a result, reliable generation of double emulsion droplets is essential for both fundamental studies and practical applications. Compared with conventional bulk emulsification techniques, microfluidics provides precise control over droplet formation through well-defined flow regimes, enabling the generation of monodisperse double emulsions with tunable core–shell sizes.

Various microfluidic geometries, including co-axial devices,^11,12^ double T-junctions,^13,14^ and flow-focusing configurations,^15,16^ have been developed for generating double emulsions. Formation strategies for double emulsions are typically divided into one-step and two-step approaches based on the droplet breakup sequence,^15^ shown in Fig. 1. In the one-step configuration (Fig. 1a), the inner, middle, and outer phases converge at a single junction, leading to simultaneous breakup of all phases. Although this design can simplify wetting treatment of devices, the strong coupling of multiphase flows often makes droplet formation highly sensitive to small perturbations in flow conditions. In contrast, the two-step approach separates the breakup process into sequential stages (Fig. 1b). First, the inner droplet forms, for example as a water-in-oil dispersion. Then, this droplet is encapsulated by the outer aqueous phase at the second junction. This staged process improves stability and allows greater flexibility in adjusting the core and shell sizes.^17^

**Fig. 1:**
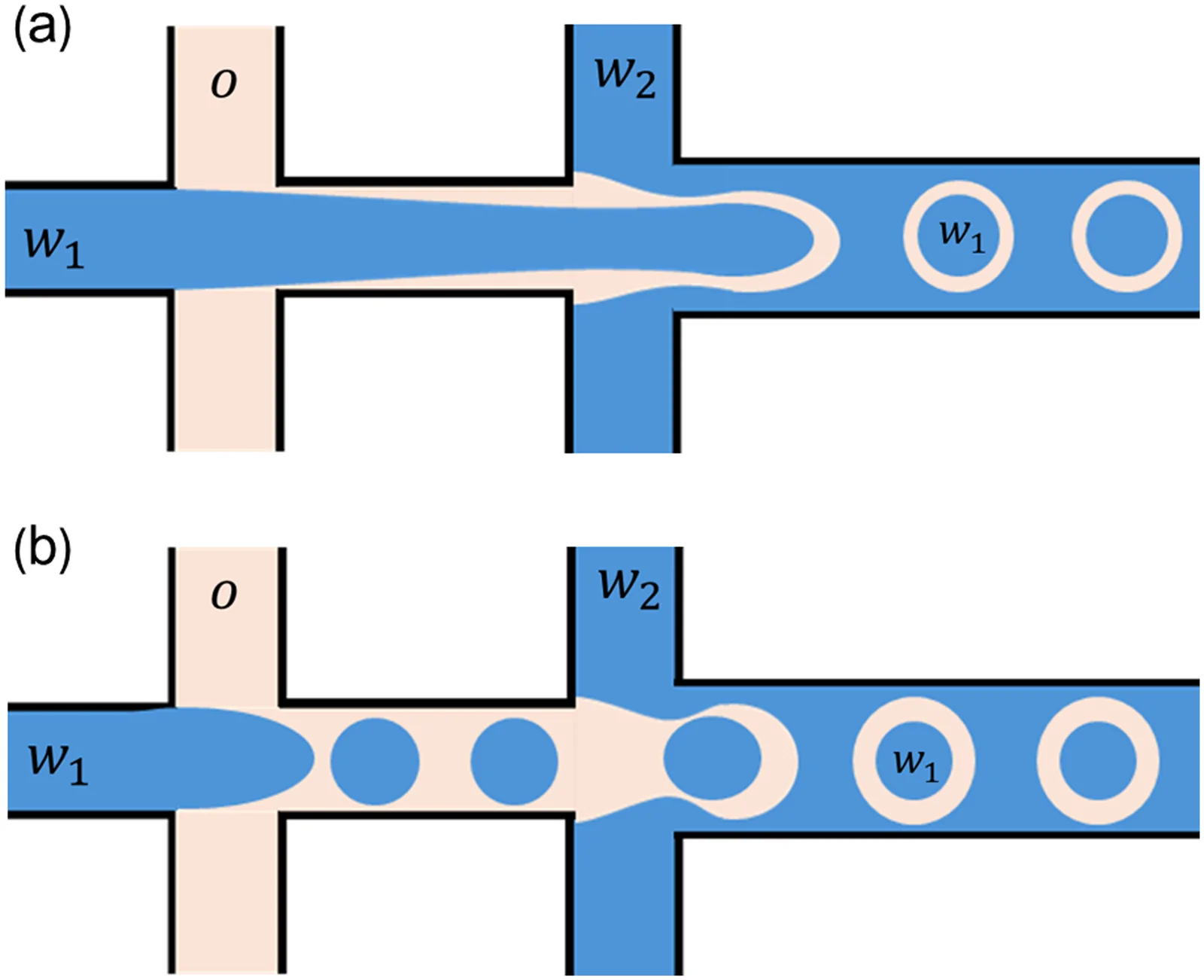
A schematic illustration of double emulsion formation: (a) one-step method and (b) two-step method.

Despite considerable progress, controlling the formation of double emulsions remains challenging. In droplet generation in microfluidic channels, common flow regimes include dripping, jetting, squeezing, and threading. Dripping occurs when interfacial tension dominates, producing discrete droplets with uniform size, whereas jetting arises under higher flow rates where inertial or viscous forces stretch the dispersed phase into a continuous thread before breakup. Squeezing is driven by pressure differences at the junction. For the more complex compound double emulsions, multiphase interactions give rise to additional patterns and transitional states. These regimes are highly sensitive to operating conditions, thus minor variation in the flow rates of the inner, middle, and outer phases can result in different flow patterns and encapsulation behavior.^18–20^ However, many existing studies have focused primarily on channel geometry or droplet size uniformity, while systematic regime mapping and size prediction for two-step double emulsion systems remain limited.^21,22^ Other investigations rely mainly on numerical simulations with minimal experimental validation.^23–26^ A systematic framework for designing encapsulated droplet generation in two-step double emulsion systems is still lacking.^27^

In this study, we adopt a two-step microfluidic platform to systematically investigate the formation of water-in-oil-in-water (w_1_/o/w_2_) encapsulated droplets over a wide range of flow conditions and interfacial tensions. A generic and well-controlled model system, including silicone oil, water/glycerol solution and sodium dodecyl sulfate (SDS), was employed to isolate the underlying physical processes. We vary the flow rates of the three phases and the interfacial tension because they are the most direct and widely used variables to control droplet formation in microfluidic applications. Using high-speed imaging, we identify and classify distinct encapsulated droplet regimes based on their shape and formation process, then construct flow pattern maps. We also develop a semi-empirical model to predict the encapsulated droplet size in the drop-in-drop configuration. The effect of dynamic interfacial tension on encapsulated droplet size is further analyzed at lower surfactant concentrations, where equilibrium interfacial tension may not be reached during droplet formation. The results provide a quantitative framework and offer design guidance for double emulsion droplet generation in microfluidic devices.

## 2 Experimental studies

### 2.1 Materials

The experiments were performed in double a cross-junction glass microchannel (Dolomite Microfluidics, UK). The junction inlet dimensions of the channels were 195 μm × 190 μm (width × depth), while the main channels had 390 μm × 190 μm (width × depth), as shown in Fig. 2a. The first section of the microchannel was hydrophobic to promote the formation of primary w_1_/o droplets, whereas the second section was hydrophilic, allowing the subsequent encapsulation step and the formation of w_1_/o/w_2_ double emulsions (Fig. 2b). Throughout the experiments, the device material, surface treatment, and surfactant type were kept the same to ensure consistent wettability conditions.

**Fig. 2:**
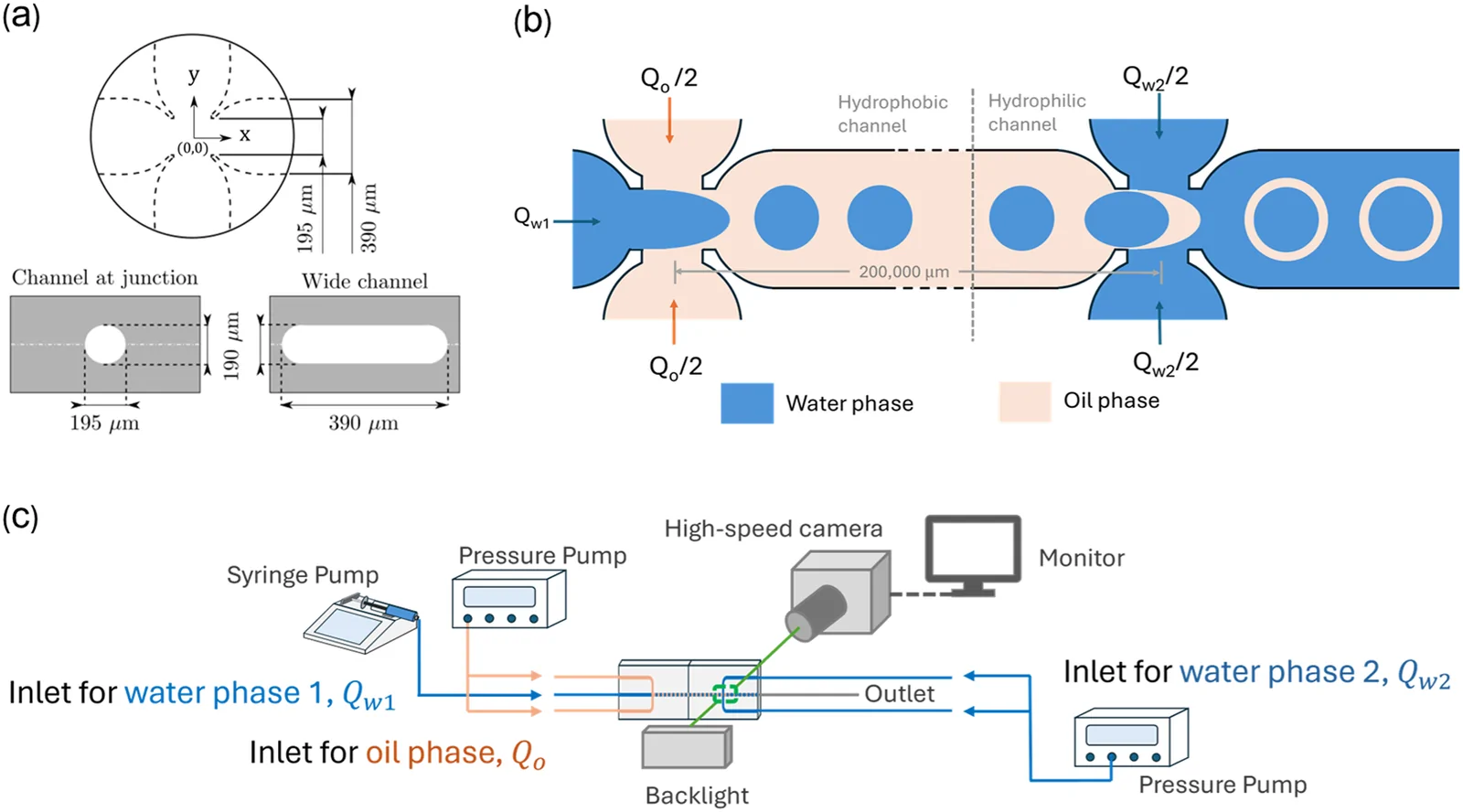
Schematics of the devices and of the experimental setup: (a) flow-focusing microchannel showing inlet dimensions, (b) schematic of the microchannel, showing two junctions separated by approximately 2 cm, which is significantly larger than the channel width, (c) experimental setup.

In all experiments, the organic phase was silicone oil (SO; density = 920 kg m^−3^, viscosity = 4.6 mPa s; Clearco). The aqueous phase was a solution of 52% w/w glycerol (Sigma-Aldrich, ≥99.5%) and 48% w/w deionized water (GS; density = 1132 kg m^−3^, viscosity = 7 mPa s), with surfactants. A 52% w/w glycerol solution was selected to match the refractive index of the silicone oil, *n*_i_ = 1.39 at 22 °C, minimizing optical distortion from reflections and allowing precise identification of interfaces.

In order to investigate the effect of interfacial tension, surfactant concentration was treated as a critical variable. Sodium dodecyl sulfate (SDS; VWR, *M*_w_ = 288.38 g mol^−1^, ≥99%) was selected as a model surfactant based on previous work.^28^ In many droplet microfluidic studies, surfactants are often used at concentrations near or above the critical micelle concentration, under which the interfacial tension is more likely to approach equilibrium during droplet formation.^29^ However, in some applications lower surfactant concentrations or alternative biocompatible surfactants are required to avoid contaminating the system.^30,31^ These systems may exhibit slower surfactant transport and adsorption kinetics, leading to non equilibrium interfacial conditions that can significantly affect droplet formation and size. To capture both regimes, SDS was added to the aqueous phase at concentrations of *C* = [0.2, 0.5, 1.0, 2.0] × CMC, where the critical micelle concentration is CMC = 11 mM.^32^ For conditions with *C*/CMC ≥ 1, the interfacial tension was assumed to have reached equilibrium, as further validated in the following sections, and was therefore used for model development. For *C*/CMC < 1, dynamic interfacial tension effects were examined to represent conditions where interfacial equilibrium may not be reached during droplet formation. A summary of the experimental parameters is presented in Table 1.

**Table 1:** Physical properties of aqueous and organic phases with varying SDS concentrations

	Organic phase (silicone oil)	Aqueous phase (52% glycerol + 48% water (w/w))				
	—	Surfactant free	0.2 CMC	0.5 CMC	1 CMC	2 CMC
*ρ* [kg m^−3^]	920	1132	1132	1132	1132	1132
*μ* [mPa s]	4.6	7	7	7	7	7
*γ*_eq_ [mN m^−1^]	—	32	18	14.6	10.7	10.7

### 2.2 Experimental set-up

The experimental setup is shown in Fig. 2c. A syringe pump (KDS Scientific®, accuracy ±5 × 10^−9^ mL min^−1^) was used to control the flow rate of the inner water phase in the first section of the microchannel (*Q*_w_1__) in the range of 3 μL min^−1^ to 30 μL min^−1^. The oil phase (middle phase) and the outer water phase were introduced using a multi-channel pressure pump (OB1 MK4, Elveflow), with total flow rates for both inlets of *Q*_o_ = 80 μL min^−1^ to 400 μL min^−1^ for the oil phase and *Q*_w_2__ = 200 μL min^−1^ to 1000 μL min^−1^ for the outer water phase. These flow rate ranges of *Q*_w_1__ and *Q*_o_ were chosen to ensure that inner droplet formation remained in the dripping regime, since this mode generates monodisperse droplets with stable and repeatable formation, which is essential for consistent encapsulation.

Droplet formation was recorded using a 12-bit high-speed camera (Phantom v1310, 1280 × 800 pixels) operating at 10 kHz to record the fast drop formation dynamics. An LED backlight was used to ensure homogeneous illumination of the channels. For each condition, at least 20 repeatable droplet formation events were captured. Images were processed in MATLAB R2023a after pixel calibration based on channel width (see SI). The inner droplets, nearly circular, were measured by contour fitting, giving the coefficient of variation (CV = standard deviation/mean droplet diameter) of 0.2–3.0%. The outer droplets were analyzed by detecting the o–w_2_ interface and calculating an equivalent diameter from the shadowgraph area, with CV values of 0.4–4.5%.

## 3 Results and discussion

### 3.1 Flow patterns

By varying the flow conditions, the jetting regime is observed in the second channel with three different patterns of double droplet formation: drop-in-drop, drop-in-plug, and drop-in-thread, as shown in Fig. 3.

**Fig. 3:**
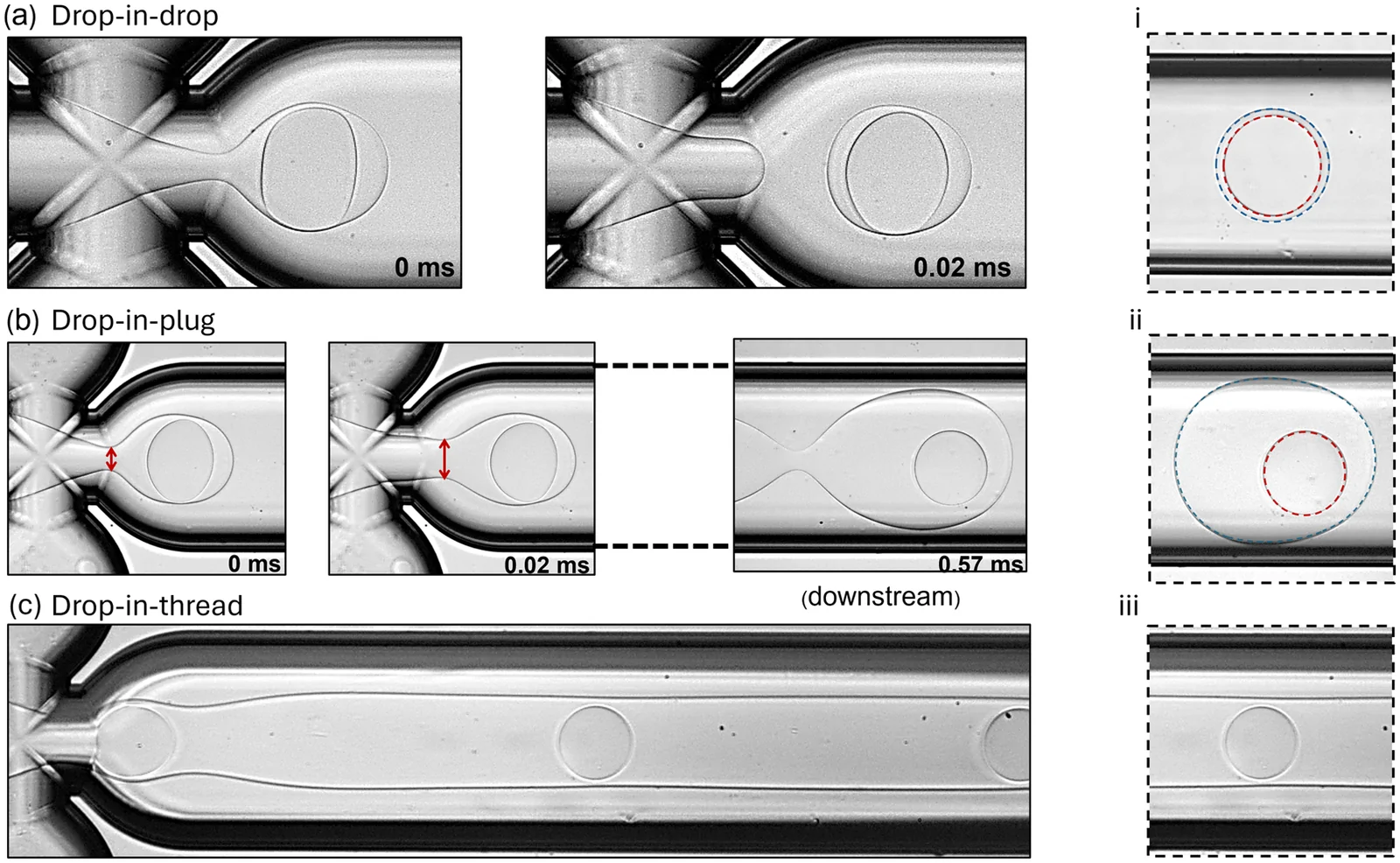
Flow patterns of double emulsion droplets. (a) i. Drop-in-drop, with the *Q*_w_1__, *Q*_o_, *Q*_w_2__ of 10, 200, 600 μL min^−1^ (0.2 CMC). In this pattern, the neck directly contracts and pinches off near the junction (0 ms). (b) ii. Drop-in-plug, with the *Q*_w_1__, *Q*_o_, *Q*_w_2__ of 12, 200, 400 μL min^−1^ (0.2 CMC). In this pattern, the neck first contracts to a minimum without breaking (0 ms), then widens and develops into an extended oil jet (0.02 ms), and finally pinches off downstream (0.57 ms). The red arrows indicate the contracted oil neck before pinch-off. (c) iii. Drop-in-thread, with the *Q*_w_1__, *Q*_o_, *Q*_w_2__ of 20, 80, 200 μL min^−1^ (1 CMC).

In the drop-in-drop pattern (Fig. 3i), once formed in the first junction, the inner droplet is carried downstream by the flow. When it enters the neck region of the oil jet in the second junction, its relatively large volume induces a large curvature in the outer o/w_2_ interface and consequently a large Laplace pressure, as illustrated in Fig. 3a. Pinch-off then occurs rapidly resulting in a spherical shell structure at 0 ms, which corresponds to the reference frame shown in Fig. 3a. As the process is fast (<3.86 ms), only a small volume of oil is released and the droplets formed have a thin shell and a concentric core–shell structure. These structural features can be relevant to applications that require well controlled double emulsion geometry, such as fluorescence-activated cell sorting (FACS)-based analysis and screening,^33^ as well as the fabrication of core–shell microcapsules, where droplet geometry, particularly shell thickness, can influence the structure and mechanical stability of the resulting capsules.^9,34^ It should be noted that these observations are obtained in a model fluid system, and their relevance to practical encapsulation systems remains to be further examined.

The drop-in-plug pattern is characterized by a thicker oil layer and smaller core droplets, as shown in Fig. 3ii. Beyond the morphological differences, the main distinction from the drop-in-drop pattern lies in the pinch-off mechanism. When a core droplet enters the second junction, the oil neck pinch-off (Fig. 3b) does not occur immediately at 0 ms, as in the drop-in-drop pattern, because the local neck curvature is not as large as in the drop-in-drop regime to induce a fast breakup. Instead, the neck region continues to move downstream (after 0.02 ms), and pinch-off eventually occurs later (at 0.57 ms). The longer formation time allows more oil to accumulate around the core droplet, resulting in a thicker shell layer. This jetting-like breakup process is more sensitive to flow instabilities, making the resulting pattern more complex and less controllable.^35^ In addition, the larger shell volume allows internal circulation, which can lead to core–shell eccentricity and displacement of the core within the shell. Although this pattern is less suitable for precise size control, it can be applied to the fabrication of multifunctional particles, such as Janus particles,^5^ formed when the inner core partially protrudes through the shell, where controlled asymmetry may be more important.

When the flow rate of the middle oil phase (*Q*_o_) is relatively large compared to the other two phases, the system transitions to the drop-in-thread pattern, as shown in (Fig. 3iii). In this regime, the oil phase no longer pinches off at the second junction, but instead extends into a continuous thread that carries the inner droplets downstream. As a result, stable double emulsion droplets are no longer formed.

### 3.2 Flow pattern maps

Maps of the observed flow patterns are presented in Fig. 4 with the three flow rates as coordinates for different surfactant concentrations. As can be seen, the imposed flow rates strongly influence the observed flow regimes. Surfactants also play a crucial role in driving the flow pattern transitions as they modify the interfacial tension. To investigate this influence, the SDS concentration was systematically varied from 2.2 mM (*C*/CMC = 0.2) to 22 mM (*C*/CMC = 2).

**Fig. 4:**
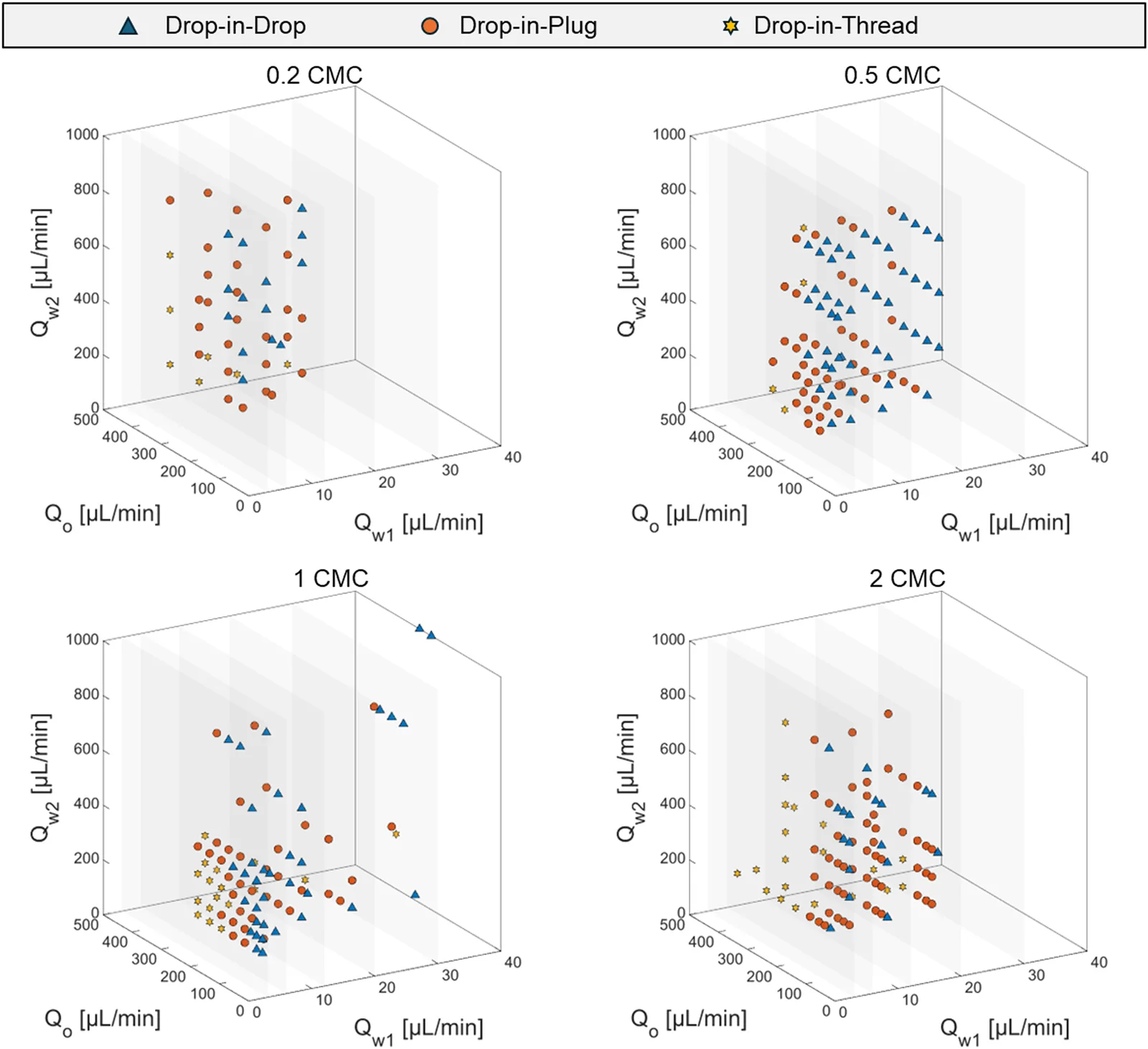
Flow pattern maps of double-emulsion formation under different flow rates and surfactant concentrations.

From the flow pattern maps in Fig. 4, it is evident that variations in the oil phase flow rate (*Q*_o_) is particularly important because it affects both the size and spacing of the inner droplets generated at the first junction and the oil neck formed at the second junction. When *Q*_o_ is relatively small and both the inner (*Q*_w_1__) and outer (*Q*_w_2__) water flow rates are comparatively large, the resulting flow pattern is drop-in-drop. In this regime, the presence of a large core and a narrow neck of the oil phase promote pinch-off near the junction, leading to the encapsulation of the inner droplet within a thin oil shell.

As the middle-phase flow rate (*Q*_o_) increases, the system gradually transitions to the drop-in-plug flow pattern as a result of two effects. The rise in *Q*_o_ leads to the formation of small core droplets at the first junction and a wide jetting neck at the second junction. In this case, the core droplet no longer dominates the pinch-off process. Instead, the core droplet is transported within the middle-phase jet, and the oil neck pinches off downstream to form a double emulsion droplet.

With a further increase in the middle-phase flow rate (*Q*_o_), especially when the outer phase flow rate (*Q*_w_2__) is also relatively high, the system transitions into the drop-in-thread pattern. In this regime, the oil phase elongates into a jetting structure dominated by inertial forces,^36^ forming continuous oil threads. Under these conditions, double emulsions are no longer produced.

The resulting three-dimensional flow pattern maps in Fig. 4 demonstrate that, in addition to flow rates, changes in surfactant concentration can significantly alter the boundaries between drop formation flow regimes. To better illustrate the influence of interfacial tension, the maps were projected onto the *Q*_o_–*Q*_w_2__ plane at *Q*_w_1__ = 12 μL min^−1^ and *C* = 0.2 CMC (Fig. 5), where dashed lines denote the boundary between drop-in-drop and drop-in-plug regimes at *C* = [0.5, 1, 2] × CMC. As the interfacial tension decreases with increasing surfactant concentration, the area of the drop-in-plug pattern in the map increases. This shift reflects a change in the balance between capillary forces and viscous stresses during droplet breakup. Consistent with the conclusions of Wu *et al.*,^37^ lower interfacial tension leads to smaller inner drops favouring the drop-in-plug pattern in the second junction. As a result, the drop-in-drop region, which corresponds to droplets with a relatively uniform shell around the core, becomes progressively narrower. These findings highlight the sensitivity of flow regimes to interfacial conditions and emphasize the role of surfactant concentration as a key parameter for the control of encapsulation. More importantly, when the surfactant concentration exceeds the CMC and the outer-phase flow rate *Q*_w_2__ is relatively low (<600 μL min^−1^), the flow pattern no longer changes with increasing concentration. This indicates that, under these conditions, the surfactant has already saturated the interface during the double droplet formation, and the interfacial tension has reached equilibrium value.

**Fig. 5:**
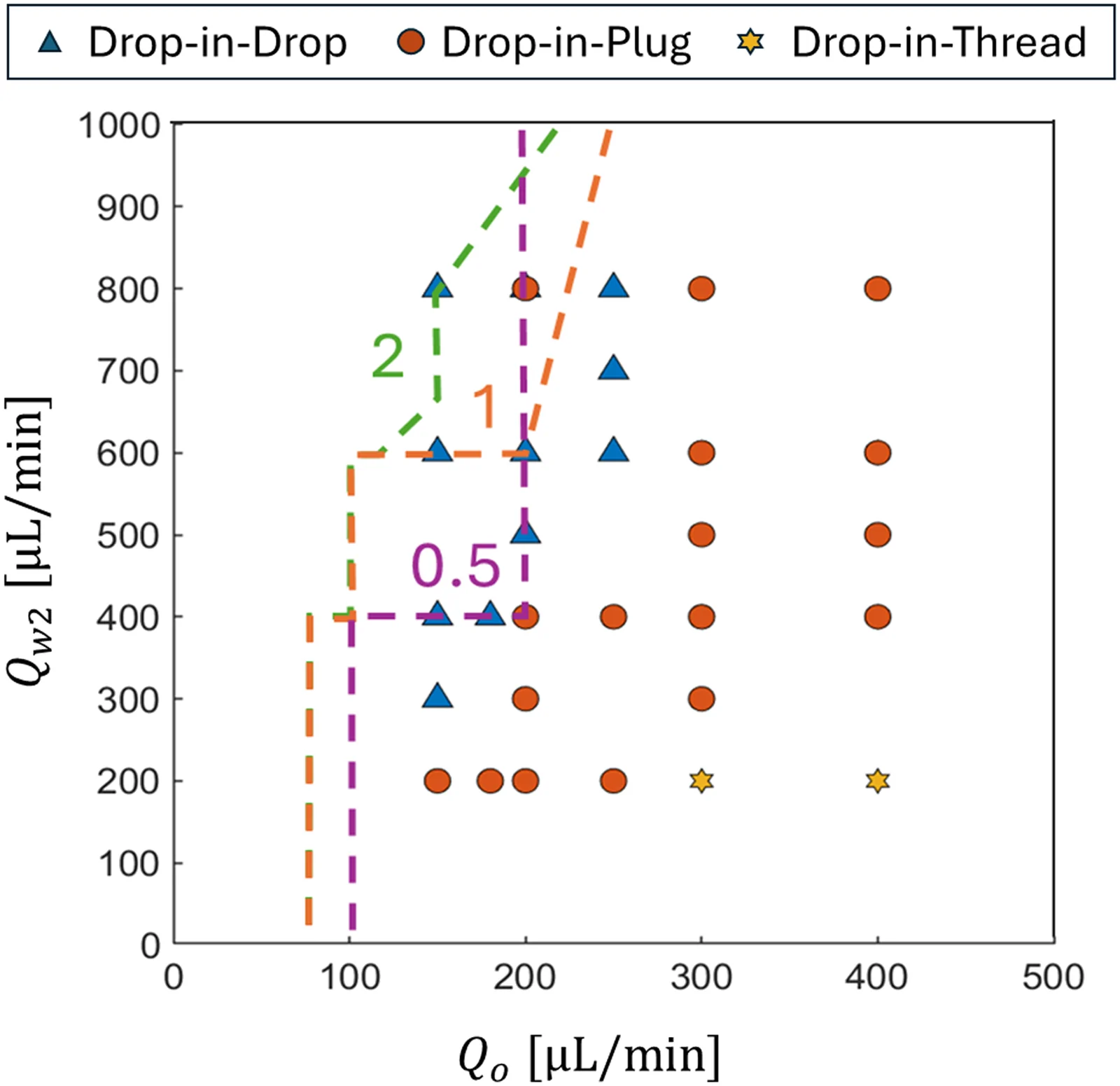
Flow pattern maps projected onto the *Q*_o_–*Q*_w_2__ plane at *Q*_w_1__ = 12 μL min^−1^. The symbols show the observed flow patterns at *C* = 0.2 CMC, while the dashed lines indicate the boundaries between drop-in-drop and drop-in-plug regimes at higher surfactant concentrations for comparison.

### 3.3 Prediction of droplet size

Since the drop-in-drop pattern produces a well-defined core–shell structure and is more suitable for applications requiring controlled encapsulation and predictable droplet geometry, a predictive model is developed for drop size in this regime based on the measurements. The results show that the encapsulated droplet size depends on the phase flow rates. In addition, as shown in Fig. 6, there is a strong correlation between the core and the outer droplet sizes.

**Fig. 6:**
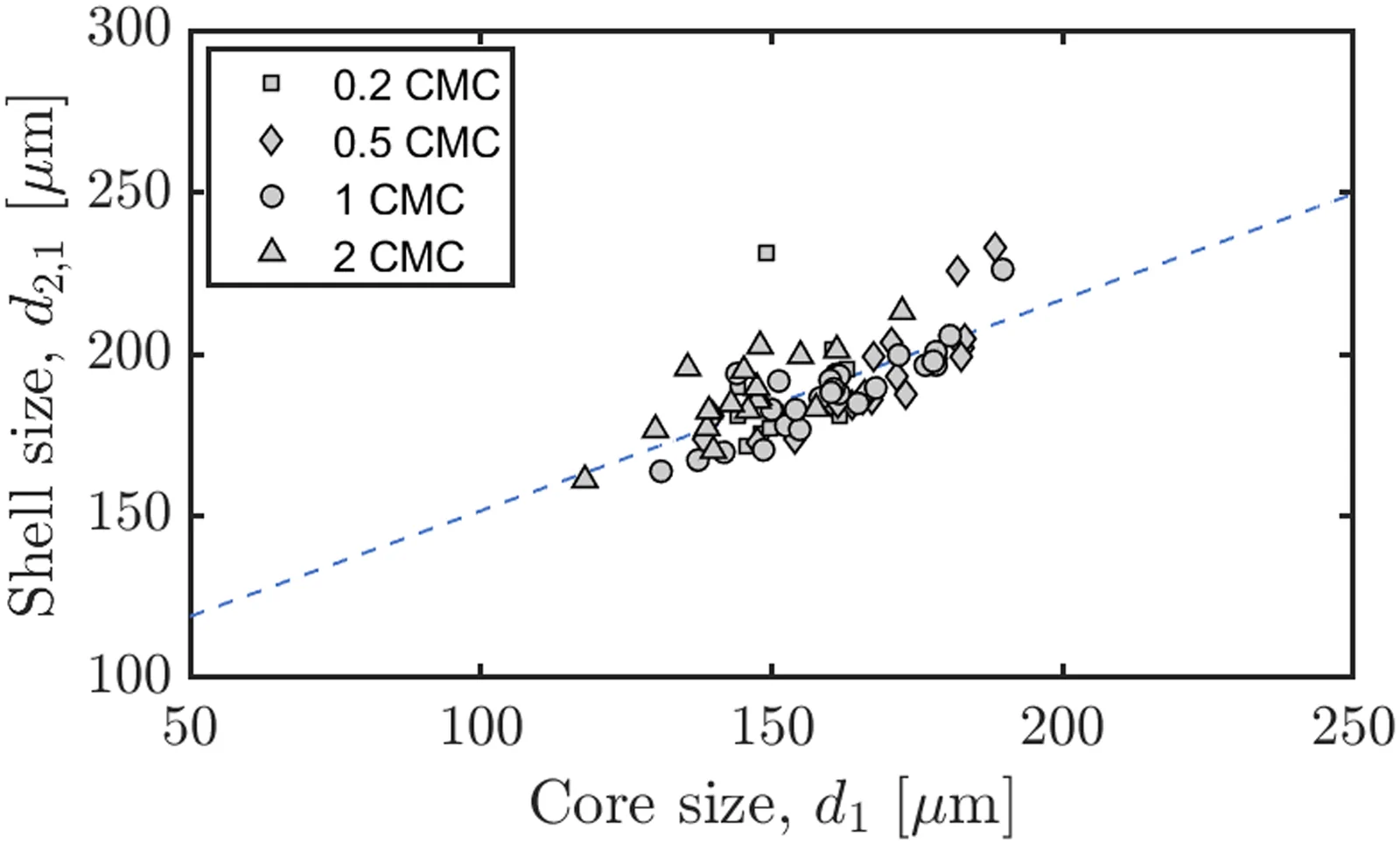
Comparison between the core droplet size and the shell droplet size in drop-in-drop regime.

Following the work of Xu *et al.*,^38^ Kalli *et al.*^32^ considered a balance between shear and capillary forces and found the following semi-empirical correlation for predicting aqueous droplet size in a continuous oil phase in a single flow-focusing channel (eqn (1)):1
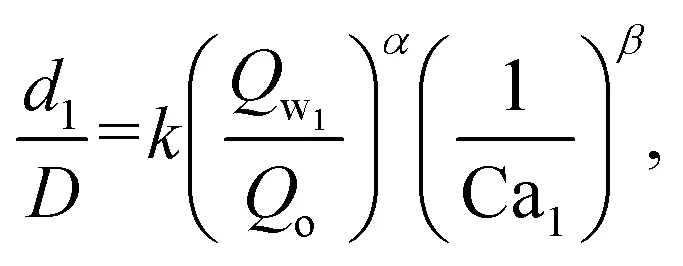
with *k* = 0.64, *α* = 0.2 and *β* = 0.167. Ca_1_ = *μ*_o_*Q*_o_/(*γA*), where *A* is the inlet cross-sectional area and *D* is the characteristic width of the flow-focusing inlet (channel width = 195 μm). This correlation was later confirmed by Chagot *et al.*^39^ using equilibrium interfacial tension on a wide range of surfactant types and concentrations to predict the droplet size with a mean absolute percentage error (MAPE) (eqn (2)) of 11.4%.2
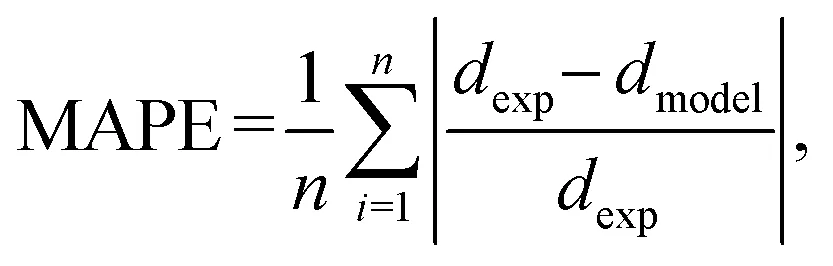


We found that the correlation, using the same parameters, also predicted our first-junction droplet size data well, indicating that the presence of the second channel junction did not affect the drop formation process in the first channel. The error (MAPE) was equal to 8.16% as shown in Fig. 7 (solid markers).

**Fig. 7:**
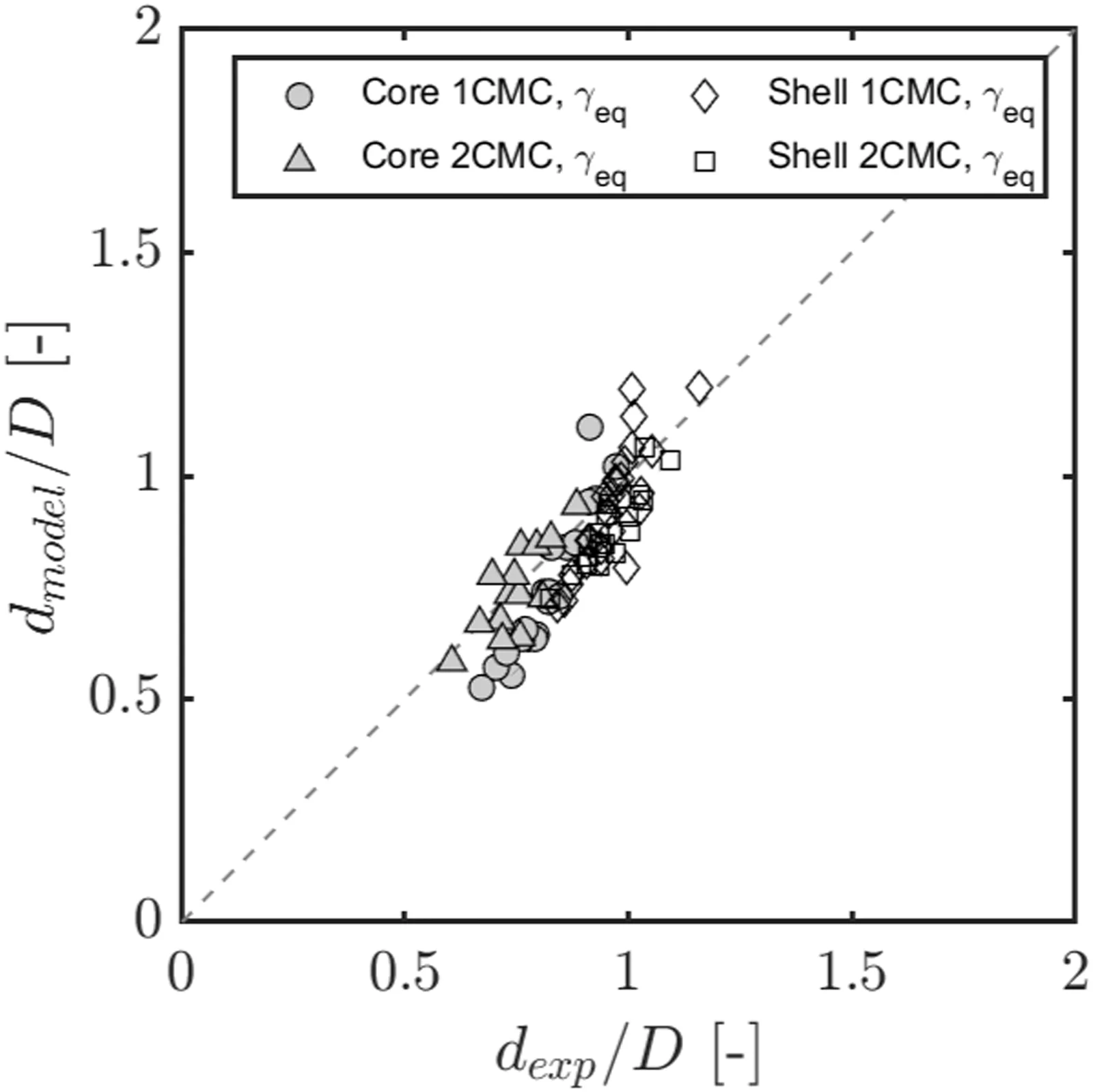
Comparison between predicted dimensionless droplet diameters and experimental data set with MAPE of 8.16% (core) and 9.54% (shell).

We now assume that, without a core droplet, a similar balance of forces determines the size of the drops (*d*_2,0_), formed in the second channel, given in the absence of encapsulation by3
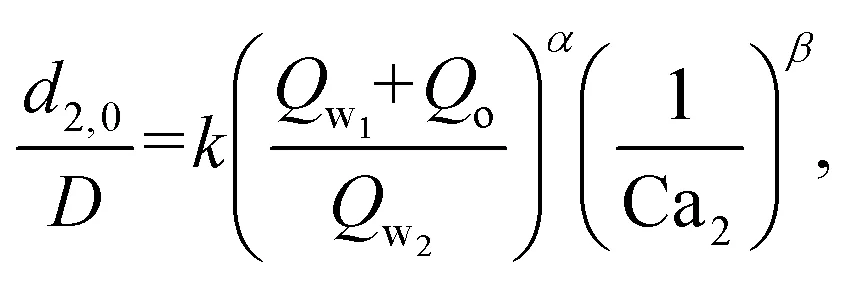
with *k* = 0.64, *α* = 0.2 and *β* = 0.167. Ca_2_ = *μ*_w_*Q*_w_2__/(*γA*).

In the present study, we focus on drop-in-drop encapsulation with shell droplet diameters *d*_2,1_ smaller than the channel width *D* (*d*_2,1_ < 195 μm), allowing us to assume nearly spherical systems.

Since it was shown in Fig. 6 that the size of the first drop significantly affects the size of the double drop, we assume that the total double droplet volume (*V*_2,1_) is the sum of the core-droplet volume (*V*_1_) and the oil-shell volume (*V*_2,0_) generated at the second junction.4
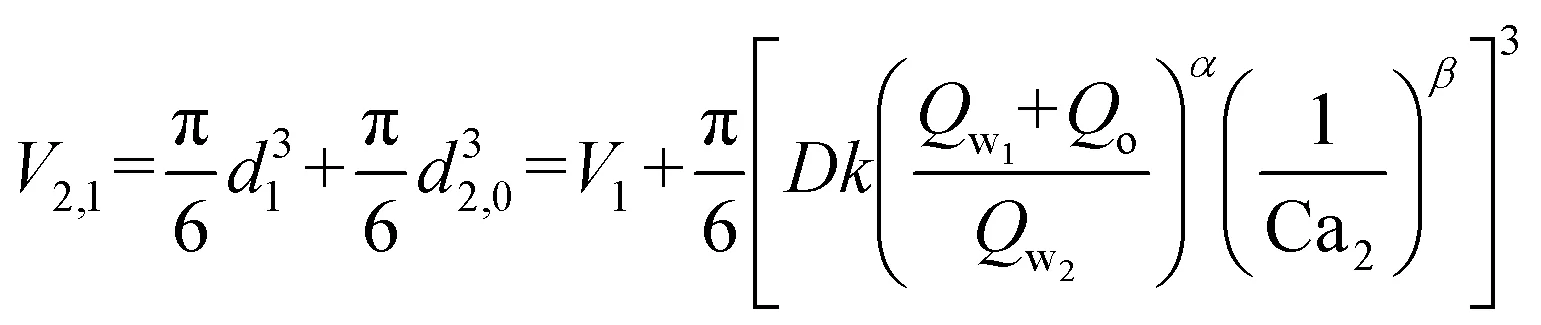
where *V*_1_ can be obtained either directly from the experimental measurements or by using eqn (1), with the same *k*, *α* and *β* previously defined.

The droplet size, *d*_2,1_, can then be recovered from the outer droplet volume as follows:5
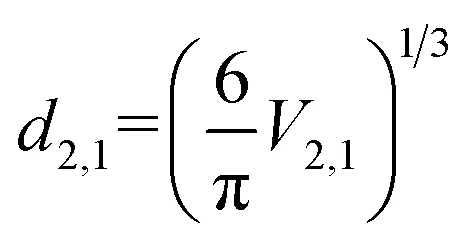
As shown in Fig. 7 (open markers), the predicted outer droplet diameter agrees well with the experimental data, with a mean absolute percentage error (MAPE) of 9.54%.

#### 3.3.1 Effects of dynamic interfacial tension (DIT)

The droplet size prediction model developed above was based on conditions where the surfactant concentration was sufficiently high for the interfacial tension to approach its equilibrium value during the formation of both core and shell droplets. However, for surfactants with slower transport rates or adsorption kinetics, or at lower concentrations, the interfacial tension may not reach its equilibrium value within the droplet formation time.^32^ Under these conditions, the dynamic interfacial tension may need to be considered. According to the approach suggested by Kalli and Angeli,^28^ the dynamic interfacial tension can be calculated from eqn (3), for the drops formed in the first junct. The dynamic interfacial tension results at different drop formation times are plotted in Fig. 8, which shows that for surfactant concentrations 0.2 and 0.5 CMC the values are higher than the equilibrium ones at short drop formation times. Under high surfactant concentrations, 1 and 2 CMC, the values are close to equilibrium ones.

**Fig. 8:**
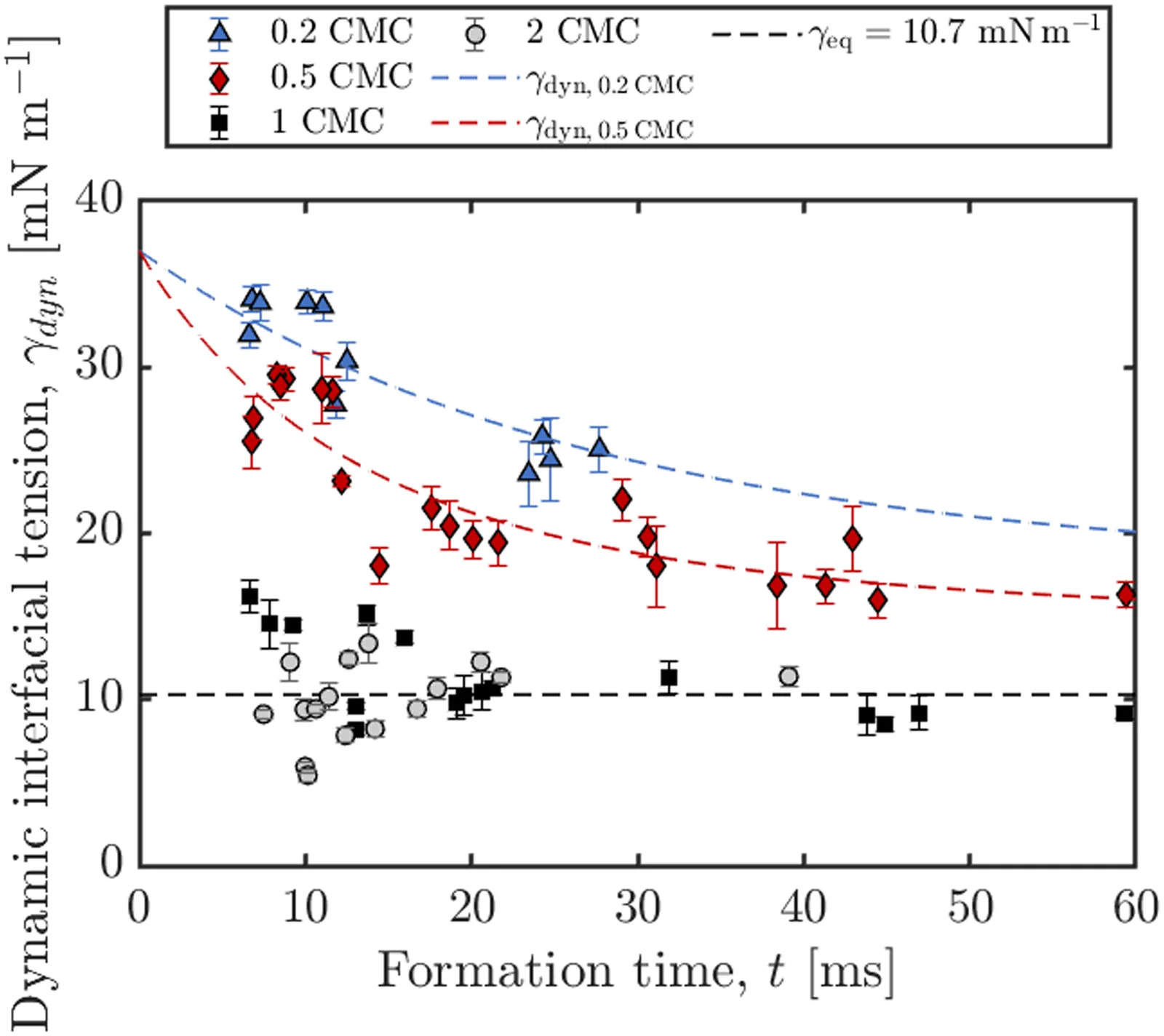
Dynamic interfacial tension against droplet formation time for different surfactant concentrations for core droplet formed in the first junction.

The dynamic interfacial tension data at 0.2 and 0.5 CMC were fitted using a constrained shifted power-law decay model that satisfies two physical limits. At zero formation time, *γ*_dyn_ was equal to 37 mN m^−1^, corresponding to the equilibrium interfacial tension of a surfactant-free interface. As the formation time approaches infinity, meaning sufficiently long formation times, *γ*_dyn_ was constrained to asymptotically approach the corresponding equilibrium value at each concentration, *γ*_eq,0.2_ = 18 mN m^−1^ and *γ*_eq,0.5_ = 14.6 mN m^−1^. Under these constraints, the remaining decay parameters were determined by nonlinear regression. Assuming that the adsorption dynamics at the second junction follow the same time-dependent trend, the DIT during the double-droplet formation was estimated from the measured formation time at each condition. These DIT values were subsequently introduced into eqn (3) and the resulting predictions were compared with those obtained using the equilibrium interfacial tension values, as shown in Fig. 9. The results show that, for 0.2 and 0.5 CMC, using the dynamic interfacial tension gives a slightly better fit (MAPE = 8.17%, magenta markers) than using the equilibrium interfacial tension, MAPE = 9.54% (green markers).

**Fig. 9:**
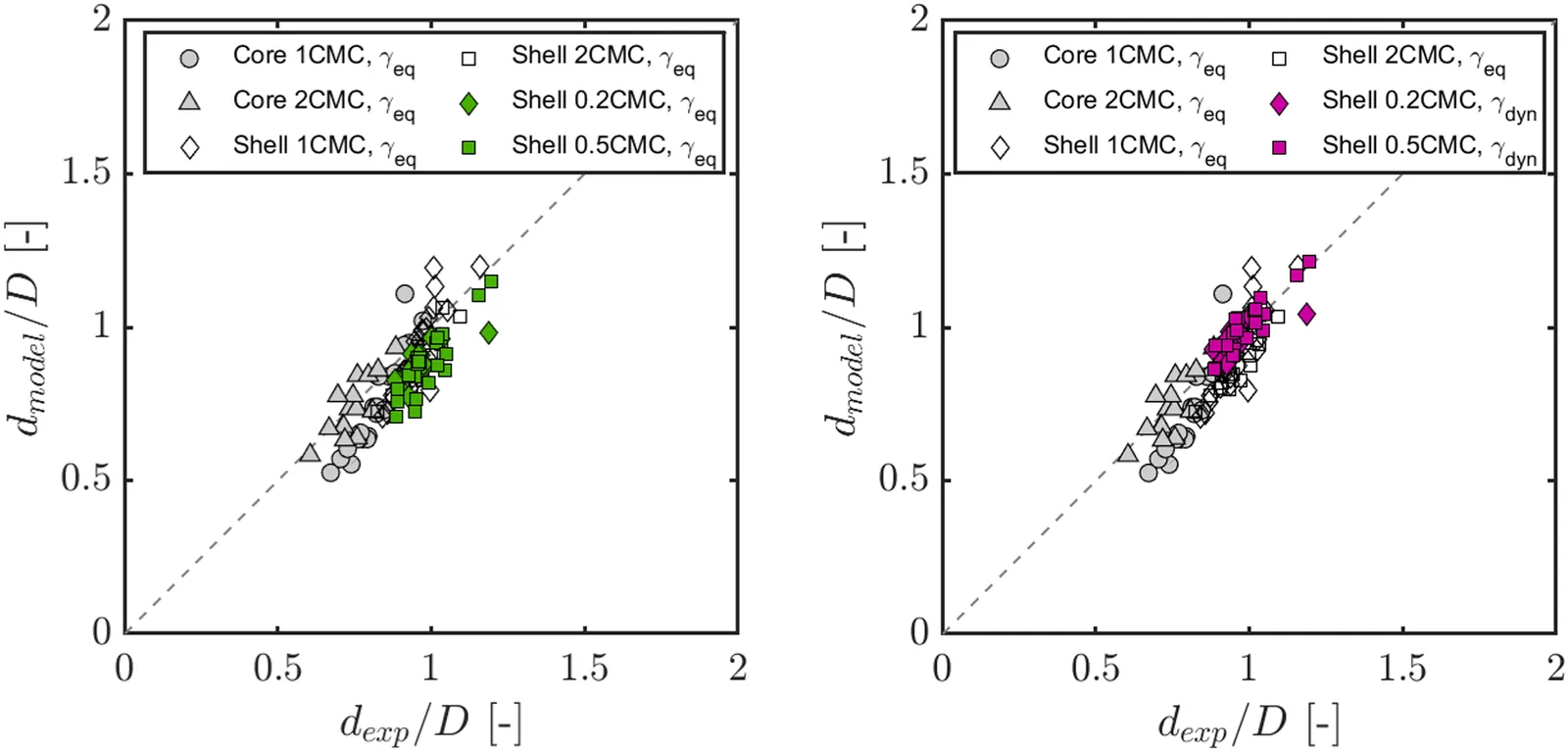
Comparison of double-droplet size predictions based on equilibrium (left) with MAPE of 9.54%; and dynamic (right) interfacial tension with MAPE of 8.17%.

## 4 Conclusions

This study systematically investigated the generation of w_1_/o/w_2_ double emulsion droplets in a two-step microfluidic system over a wide range of flow rates and interfacial conditions using a generic model system. By varying the phase flow rates and the concentration of SDS, three distinct double-drop formation patterns were identified, namely drop-in-drop, drop-in-plug and drop-in-thread. Based on these observations, a flow pattern map was established, providing an operating window for the controlled generation of different droplet patterns. Among them, the drop-in-drop pattern is of particular practical relevance for encapsulation applications and was thus selected for developing a predictive model for droplet size.

Semi-empirical correlations were developed to predict both the core droplet and the overall double droplet sizes, with errors of 8.16% and 9.54% respectively. In addition, the effects of dynamic interfacial tension and equilibrium interfacial tension on double droplet size prediction were compared at low surfactant concentrations. The small reduction in MAPE from 9.54% to 8.17% suggests that, although DIT has a measurable influence at low surfactant concentrations, the equilibrium values are sufficient to provide good estimations of both inner and outer droplet sizes for the surfactant used here.

Beyond these specific findings, this study advances the fundamental understanding of the underlying physics, and the resulting framework can help inform the design of applications such as bio-encapsulation systems. However, the present work is limited to the range of flow rates and interfacial conditions considered here, as well as to the model system adopted. Therefore, the applicability of the results to more complex fluids and practical applications still needs to be examined. Future work will extend the present analysis to a wider class of analogous systems and practical configurations. Moreover, correlations for the drop-in-plug pattern will be developed over a broader range of flow and interfacial conditions. The flow fields in forming single and double droplets will also be investigated to clarify the effects of internal circulation patterns on interface deformation and breakup. Such efforts are expected to provide deeper mechanistic insight and to support the rational design and optimization of droplet-based encapsulation systems.

## Author contributions

Chen Tang: data curation, conceptualization, investigation, validation, software, writing – original draft preparation/reviewing and editing, visualization. Loïc Chagot: methodology, software, writing – original draft preparation/reviewing and editing, visualization, investigation. Panagiota Angeli: supervision, writing – reviewing and editing, funding acquisition, resources, conceptualization.

## Conflicts of interest

There are no conflicts declare.

## Supplementary Material

LC-026-D6LC00406G-s001

LC-026-D6LC00406G-s002

## Data Availability

The data supporting this article have been included as part of the supplementary information (SI). Supplementary information: SI figures. See DOI: https://doi.org/10.1039/d6lc00406g.
