# Supplementary material for: Controlled encapsulation and droplet size prediction in two-step microfluidic double emulsions
Source: Lab Chip. 2026 Jun 24;26(19):5232–40. doi: 10.1039/d6lc00406g (PMC13623284; doi:10.1039/d6lc00406g)

Supplementary Information (SI) for Lab on a Chip.
This journal is © The Royal Society of Chemistry 2026

Core-droplet identification

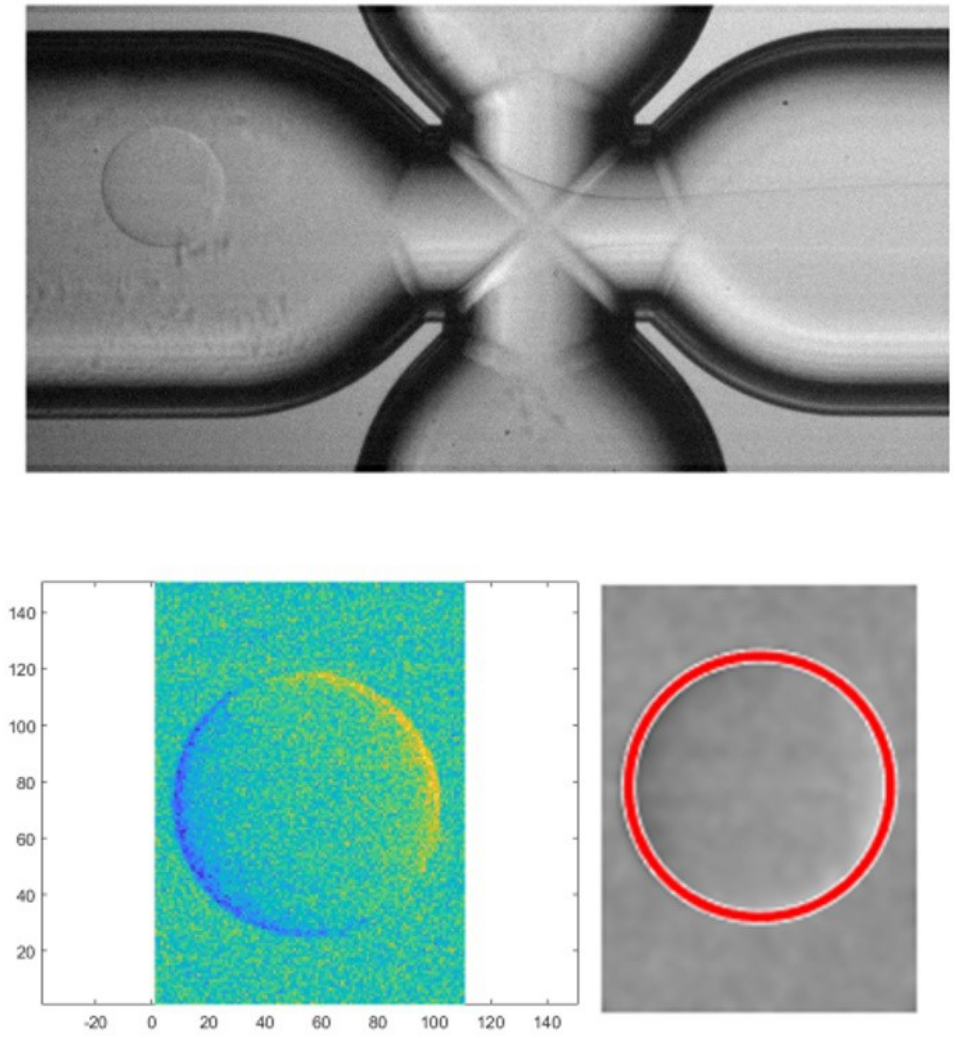

Shell-droplet identification

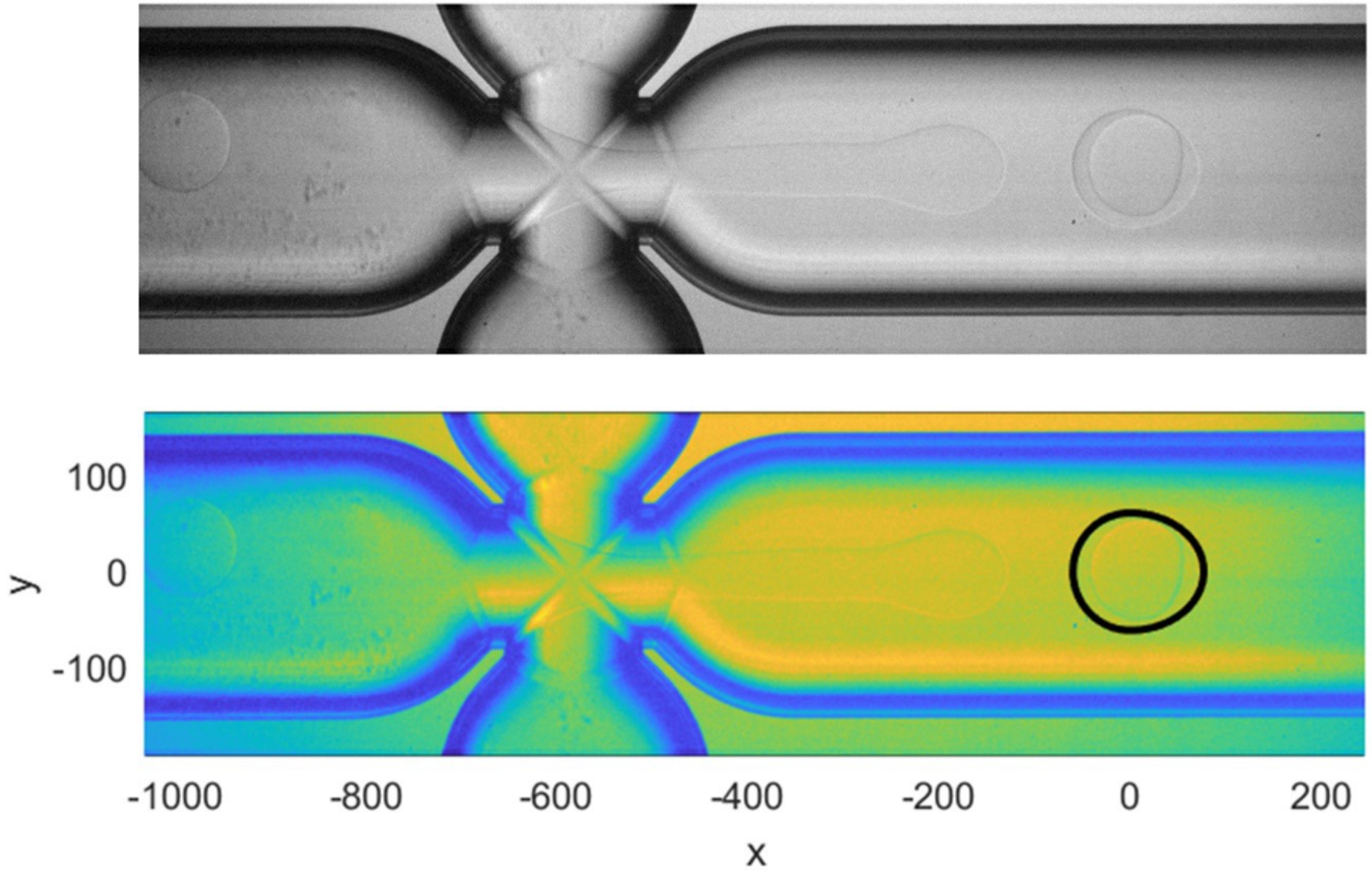

Supplement: Supplementary file 1 [file LC-026-D6LC00406G-s001.pdf]
